# Prognostic value of the pre-treatment SUVmax of ^18^F-FDG PET/CT combined with peripheral absolute lymphocyte in patients with newly diagnosed extranodal natural killer/T-cell lymphoma

**DOI:** 10.1186/s40644-025-00882-0

**Published:** 2025-06-04

**Authors:** Xingmei Lu, Kate Huang, Peng Li, Yida Li, Xiuhuan Ji, Suidan Chen, Jianmin Li

**Affiliations:** 1 Department of Pathology, The First Affiliated Hospital of Wenzhou Medical University, Wenzhou, 325000 Zhejiang China; 2 Wenzhou Key Laboratory of Basic Science and Translational Research of Radiation Oncology, Zhejiang Engineering Research Center for Innovation and Application of Intelligent Radiotherapy Technology, Wenzhou, 325000 Zhejiang China

**Keywords:** Extranodal natural killer/T-cell lymphoma (ENKTCL), ^18^F-fluorodeoxyglucose (^18^F-FDG), Positron emission tomography/Computed tomography (PET/CT), Maximum standardized uptake value (SUVmax), Absolute lymphocyte count (ALC)

## Abstract

**Background:**

Accurate assessment and prediction of patient prognosis, early identification of high-risk patients, and improvement of clinical outcomes for individuals with extranodal natural killer/T-cell lymphoma (ENKTCL) are critical. This study evaluates the prognostic value of a novel model combining maximum standardized uptake value (SUVmax) and absolute lymphocyte count (ALC) in ENKTCL patients.

**Methods:**

We conducted a retrospective analysis of clinical data from 57 patients diagnosed with primary ENKTCL. Optimal cut-off values for SUVmax and ALC were determined using receiver operating characteristic (ROC) curves. Clinical characteristics were analyzed by Chi-squared tests or Fisher’s exact tests. Survival analysis was performed using the Kaplan-Meier method and log-rank test, while independent prognostic factors were identified through Cox regression analysis.

**Results:**

The optimal cut-off values for SUVmax and ALC were established at 11.8 and 0.87 × 10^9^/L, respectively. Univariate and multivariate analyses confirmed that both SUVmax and ALC were independent predictors of prognosis in ENKTCL patients. According to the combined SUVmax-ALC model, the patients were stratified into low-risk, intermediate-risk and high-risk groups. Kaplan-Meier analysis revealed significant differences in overall survival (OS) and progression-free survival (PFS) among these groups (*p* < 0.001). ROC curve analysis showed that the area under the curve (AUC) for the SUVmax-ALC model was 0.714, superior to individual tests (SUVmax, AUC = 0.674; ALC, AUC = 0.589). In addition, the AUC of the SUVmax-ALC model was higher than the International Prognostic Index (IPI, AUC = 0.632), nomogram-revised risk index (NRI, AUC = 0.566), and prognostic index of natural killer T-cell lymphoma (PINK, AUC = 0.592). Furthermore, the SUVmax-ALC model more effectively identified high-risk patients within low-risk IPI, PINK, or NRI groups, providing additional prognostic information. These findings indicate that the combination of SUVmax and ALC offers enhanced predictive accuracy for ENKTCL prognosis.

**Conclusion:**

Pre-treatment SUVmax and ALC can serve as valuable indicators for predicting the prognosis of ENKTCL patients. Compared to IPI, NRI, and PINK scores, the SUVmax-ALC model demonstrates superior performance in risk stratification, suggesting its potential as an effective personalized prognostic tool for ENKTCL patients.

## Background

Extranodal natural killer/T-cell lymphoma (ENKTCL) represents a rare and distinct form of non-Hodgkin lymphoma (NHL) [1, 2]. This condition exhibits a geographic distribution, being more prevalent in Asia and South America [3]. The upper aerodigestive tract (UADT), especially the nasal and paranasal regions, constitutes approximately 80% of ENKTCL cases [4, 5]. Other affected sites include the skin, gastrointestinal tract, testicles, muscles, lungs, and salivary glands [6]. No standardized therapeutic protocol exists for ENKTCL; however, radiotherapy or combined chemoradiotherapy is predominantly used for early-stage (I/II) disease, whereas multi-drug combined chemotherapy is primary approach for advanced-stage (III/IV) disease. Recent advancements in pegaspargase/l-asparaginase-based chemotherapy have markedly enhanced patient outcomes in ENKTCL [7, 8]. Despite these improvements, ENKTCL remains highly malignant, aggressive, and rapidly progressive, leading to significant recurrence rates and poor survival outcomes in many patients [9]. Consequently, precise evaluation and prediction of patient prognosis, along with the early identification of high-risk individuals, are essential for optimizing clinical management and promoting personalized precision medicine.

To date, the International Prognostic Index (IPI) and the prognostic index of natural killer T-cell lymphoma (PINK) have been developed and applied to predict the prognosis of ENKTCL [10, 11]. Nonetheless, the predictive accuracy of these indices in ENKTCL has been found to be limited [12]. In comparison, the nomogram-revised risk index (NRI) has demonstrated superior performance in predicting overall survival (OS) [13].

In recent years, the significance of ^18^F-fluorodeoxyglucose positron emission tomography/computed tomography (^18^F-FDG PET/CT) in lymphoma prognosis, treatment response assessment, early diagnosis, and initial staging has gained widespread recognition, particularly in Hodgkin lymphoma and diffuse large B cell lymphoma (DLBCL) [14, 15]. Advances in software and image processing have heightened the interest in the prognostic value of PET/CT metabolic parameters, such as maximum standardized uptake value (SUVmax), metabolic tumor volume (MTV), and total lesion glycolysis (TLG). SUVmax is the most frequently examined of these parameters [16]. Given the rarity of ENKTCL, research on the predictive utility of these metabolic parameters in pre-treatment ENKTCL has been limited, with inconsistent findings [17–23]. Further investigation is warranted to assess the predictive role of pre-treatment PET/CT metabolic parameters in ENKTCL risk stratification.

Immunodeficiency has been implicated in the pathogenesis and progression of lymphoma [24]. Lymphocyte-mediated antibody-dependent cell-mediated cytotoxicity serves as an indicator of host immune function [25]. Previous studies have identified pre-treatment absolute lymphocyte count (ALC) as an independent predictor of ENKTCL prognosis [26].

Exploring the combined application of SUVmax and ALC is expected to further comprehensively evaluate the prognosis of ENKTCL patients from the aspects of oncology and immunology and improve their survival rate. This study aimed to investigate the potential predictive value of SUVmax combined with ALC in ENKTCL, stratify patients risk, and provide a basis for clinical individualized and precise treatment.

## Materials and methods

### Patients

This study retrospectively analyzed clinical data from the clinical database of the First Affiliated Hospital of Wenzhou Medical University. Patients newly diagnosed with ENKTCL according to the World Health Organization (WHO) classification criteria between November 2016 and April 2023 were included. Inclusion criteria were as follows: (1) newly diagnosed with ENKTCL, (2) availability of pre-treatment ^18^F-FDG PET/CT and complete blood cell count. Exclusion criteria included prior antitumor therapy, presence of another malignant disease, or complete resection of the lesion before the first imaging examination.

For all eligible patients, baseline clinical characteristics were recorded, including gender, age at diagnosis, primary site, Eastern Cooperative Oncology Group (ECOG) performance status, Ann Arbor stage, B symptoms, lymph node status, Epstein-Barr virus (EBV) DNA titer, serum albumin level, lactate dehydrogenase (LDH) level, and complete blood cell count. Staging was performed according to the Ann Arbor staging system, and risk stratification was conducted using the IPI, PINK, and NRI models [13, 27]. All included patients underwent pre-treatment ^18^F-FDG PET/CT examination. Among them, 14 patients completed interim PET/CT examinations (after 3 to 6 cycles of chemotherapy), and 7 patients underwent end-of-treatment PET/CT assessments following first-line therapy. For patients with multiple lesions, the lesion with the highest SUVmax at diagnosis was selected for analysis. This study was approved by the Ethics Committee for Clinical Research (ECCR) of the First Affiliated Hospital of Wenzhou Medical University. The ECCR waived the requirement for informed consent due to the retrospective nature of the study (No. KY2021-R081).

### ^18^F-FDG PET/CT image acquisition and analysis

All patients fasted for at least 6 h, ensuring blood glucose levels were below 110 mg/dL, before receiving an intravenous injection of 3.7 MBq/kg of ^18^F-FDG, PET/CT images were acquired 60 min post-injection using a hybrid PET/CT scanner (Gemini 64 TF, Philips, The Netherlands). For accurate anatomical localization and attenuation correction, a low-dose unenhanced CT scan was performed from the base of the skull to the mid-thigh with the following parameters: 120 kV, 80 mA, a pitch of 0.829, a tube rotation speed of 0.5 s, and a reconstruction thickness and interval of 5.0 mm. A three-dimensional mode PET scan matching the CT segment thickness followed. The ordered subset expectation maximization method was used to reconstruct the PET images. All acquired images were displayed on the Philips Extended Brilliance Workstation (EBW) 3.0 for PET, CT, and PET/CT fusion image reconstruction. Two experienced nuclear medicine physicians analyzed the images using a dedicated workstation (Philips EBW 3.0). The volume of interest (VOI) was automatically delineated using the threshold SUV method, and the highest voxel within the VOI was identified as the SUVmax.

### Statistical analysis

Statistical analysis was performed using SPSS version 26.0. To evaluate the optimal cut-off values for SUVmax and ALC to predict OS, we performed receiver operating characteristic (ROC) curve analysis to determine the cut-off values using the Youden Index to maximize specificity and sensitivity. Then, the patients were divided into two SUVmax groups and two ALC groups by the cut-off values. Chi-square tests or Fisher’s exact tests were used to compare the clinical characteristics of these groups. Progression-free survival (PFS) was defined as the time from initial diagnosis to disease progression, death from any cause, or the last follow-up for surviving patients. OS was defined as the time from initial diagnosis to death from any cause or the last follow-up visit. Kaplan-Meier analysis was used to generate survival curves, and the log-rank test was used to compare the survival differences among patients. Univariate and multivariate Cox proportional hazards regression models were employed for survival analysis. Statistical significance was set at *p* < 0.05.

## Results

### Patient characteristics

A total of 57 ENKTCL patients met the inclusion criteria, comprising 44 males and 13 females (male-to-female ratio of 3.38:1). The patients’ clinical characteristics are listed in Table 1. The median age at diagnosis was 55 years, ranging from 24 to 82 years, with 21 patients (36.8%) aged over 60 years. Thirty-nine (68.4%) patients presented with early-stage disease (Ann Arbor stages I or II), and the upper aerodigestive tract was the most common primary lesion site (*n* = 42, 73.7%). B symptoms were present in 21 (36.8%) patients at presentation. Distant lymph node involvement was observed in 28.1% (16/57) of the patients. Elevated LDH levels were noted in about half of the patients (55.6%). The majority of patients were classified as low-risk based on the IPI (*n* = 43, 75.4%), PINK (*n* = 40, 70.2%), and NRI (*n* = 35, 61.4%) scores.

**Table 1:** Baseline patient characteristics

Characteristics	Values
Gender, n(%)	
male	44 (77.2)
female	13 (22.8)
Age, n(%)	
≤ 60	36 (63.2)
> 60	21 (36.8)
IPI score, n(%)	
≤ 2	43 (75.4)
> 2	14 (24.6)
NRI score, n(%)	
≤ 2	35 (61.4)
> 2	22 (38.6)
PINK score, n(%)	
< 2	40 (70.2)
≥ 2	17 (29.8)
ECOG PS, n(%)	
< 2	50 (87.7)
≥ 2	7 (12.3)
Ann Arbor stage, n(%)	
I/II	39 (68.4)
III/IV	18 (31.6)
B symptoms, n(%)	
absent	36 (63.2)
present	21 (36.8)
Primary site, n(%)	
UADT	42 (73.7)
EUADT	15 (26.3)
Distant lymph node involvement, n(%)	
yes	16 (28.1)
no	41 (71.9)
Albumin (g/L), n(%)^a^	
< 35	25 (46.3)
≥ 35	29 (53.7)
Blood EBV-DNA, n(%)^b^	
positive	32 (66.7)
negative	16 (33.3)
LDH, n(%)^c^	
normal	24 (44.4)
elevated	30 (55.6)
Ki−67, n(%)^d^	
< 70	31 (57.4)
≥ 70	23 (42.6)
SUVmax, median (range)	10.6 (2.9, 27.7)
ALC (× 10^9^/L), median (range)	1.17 (0.28, 4.26)

^a^Data of pre-treatment albumin were available in 54 patients; ^b^Data of pre-treatment EBV-DNA were available in 48 patients; ^c^Data of pre-treatment LDH were available in 54 patients; ^d^Data of Ki-67 were available in 54 patients

IPI, International Prognostic Index; PINK, prognostic index of natural killer lymphoma; NRI, nomogram-revised risk index; ECOG, Eastern Cooperative Oncology Group; PS, performance status; UADT, upper aerodigestive tract; EUADT, extra-upper aerodigestive tract; LDH, lactate dehydrogenase; EBV-DNA, Epstein-Barr virus DNA; SUVmax, maximum standardized uptake value; ALC, absolute lymphocyte count

Regarding the first-line treatment modality, 40 patients received chemotherapy alone, and 11 patients received chemotherapy combined with radiotherapy, 1 patient received supportive care only, and treatment information was unavailable for 5 patients. Chemotherapy regimens included: P-GEMOX (pegaspargase, gemcitabine, oxaliplatin, *n* = 33), GEMOX (gemcitabine, oxaliplatin, *n* = 4), GELOX (gemcitabine, l-asparaginase, oxaliplatin, *n* = 2), CHOP (cyclophosphamide, doxorubicin, vincristine, prednisone, *n* = 2), L-GDP (l-asparaginase, gemcitabine, dexamethasone, cisplatin, *n* = 1), P-GDP (pegaspargase, gemcitabine, dexamethasone, cisplatin, *n* = 1), SMILE (dexamethasone, methotrexate, ifosfamide, L-asparaginase, etoposide, *n* = 1), l-asparaginase combined EPOCH (etoposide, doxorubicin, vincristine, cyclophosphamide, prednisone, *n* = 1), and others (*n* = 6). Of these patients, thirty-nine (76.5%) were treated with pegaspargase/l-asparaginase-based modalities.

### Relationship between pre-treatment SUVmax and ALC levels and clinical characteristics

**Fig. 1:**
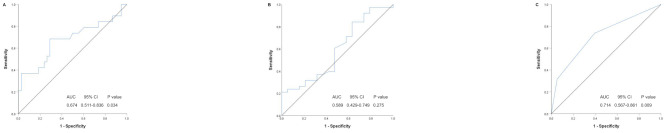
ROC curves for the discrimination between patients with SUVmax (**A**), ALC (**B**), and SUVmax-ALC (**C**). ROC, receiver operating characteristics; SUVmax, maximum standardized uptake value; ALC, absolute lymphocyte count

After performing calculations using the ROC curve in terms of the OS, we found that the optimal cut-off value for SUVmax was 11.80 [area under the curve (AUC) 0.674, *p* = 0.034] (Fig. 1A). Based on this result, patients were divided into a low SUVmax group (≤ 11.80, *n* = 33) and a high SUVmax group (> 11.80, *n* = 24). As shown in Table 2, SUVmax > 11.80 was significantly associated with a high IPI score (*p* = 0.001), a high NRI score (*p* = 0.009), advanced Ann Arbor stage (*p* = 0.011), elevated LDH level (*p* = 0.043), and a high Ki-67 index (*p* = 0.044). However, there were no significant differences in pre-treatment SUVmax among B symptoms, primary site, and blood EBV-DNA load (Table 2).

**Table 2:** Relationships between SUVmax, ALC, and SUVmax-ALC and clinical characteristics

Characteristics	SUVmax				ALC (× 10^9^/L)				SUVmax-ALC			
	≤ 11.80	> 11.80	*P* value		≤ 0.87	> 0.87	*P* value		Low-risk	Intermediate-risk	High-risk	*P* value
Gender			0.114				0.710					0.255
male	23	21			11	33			19	18	7	
female	10	3			2	11			9	3	1	
Age			0.306				1.000					0.102
≤ 60	19	17			8	28			15	17	4	
> 60	17	7			5	16			13	4	4	
IPI score			0.001				0.271					0.009
≤ 2	30	13			8	35			26	13	4	
> 2	3	11			5	9			2	8	4	
NRI score			0.009				0.524					0.089
≤ 2	25	10			7	28			21	11	3	
> 2	8	14			6	16			7	10	5	
PINK score			0.096				0.734					0.719
< 2	26	14			10	30			21	14	5	
≥ 2	7	10			3	14			7	7	3	
ECOG PS			0.119				0.333					0.062
<2	31	19			10	40			26	19	5	
≥2	2	5			3	4			2	2	3	
Ann Arbor stage			0.011				0.735					0.085
I/II	27	12			8	31			23	12	4	
III/IV	6	12			5	13			5	9	4	
B symptoms			0.230				0.051					0.051
Absent	23	13			5	31			20	14	2	
Present	10	11			8	13			8	7	6	
Primary site			0.847				1.000					0.510
UADT	24	18			10	32			21	14	7	
EUADT	9	6			3	12			7	7	1	
Distant lymph node involvement			0.110				1.000					0.162
yes	5	11			4	12			5	7	4	
no	28	13			9	32			23	14	4	
Albumin (g/L)^a^			0.583				0.206					0.450
< 35	13	12			8	17			10	10	5	
≥ 35	18	11			5	24			16	10	3	
Blood EBV-DNA^b^			0.152				0.040					0.063
positive	15	17			11	21			11	14	7	
negative	11	5			1	15			10	6	0	
LDH^c^			0.043				0.255					0.096
normal	17	7			4	20			14	9	1	
elevated	13	17			9	21			11	12	7	
Ki−67^d^			0.044				0.516					0.090
< 70	18	7			5	20			16	6	3	
≥ 70	13	16			8	21			10	14	5	

^a^Data of pre-treatment albumin were available in 54 patients; ^b^Data of pre-treatment EBV-DNA were available in 48 patients; ^c^Data of pre-treatment LDH were available in 54 patients; ^d^Data of Ki-67 were available in 54 patients

IPI, International Prognostic Index; PINK, prognostic index of natural killer lymphoma; NRI, nomogram-revised risk index; ECOG, Eastern Cooperative Oncology Group; PS, performance status; UADT, upper aerodigestive tract; EUADT, extra-upper aerodigestive tract; LDH, lactate dehydrogenase; EBV-DNA, Epstein-Barr virus DNA; SUVmax, maximum standardized uptake value; ALC, absolute lymphocyte count

The ROC curve analysis determined the optimal cut-off value for ALC to be 0.87 × 10^9^/L (AUC 0.589, *p* = 0.275) (Fig. 1B). Consequently, patients were classified into a low ALC group (≤ 0.87 × 10^9^/L, *n* = 13) and a high ALC group (> 0.87 × 10^9^/L, *n* = 44). Chi-square (χ^2^) tests revealed that low ALC was significantly associated with a high EBV-DNA load (*p* = 0.040), and showed a trend towards correlation with B symptoms (*p* = 0.051) (Table 2).

### Survival analysis and prognosis factors

**Fig. 2:**
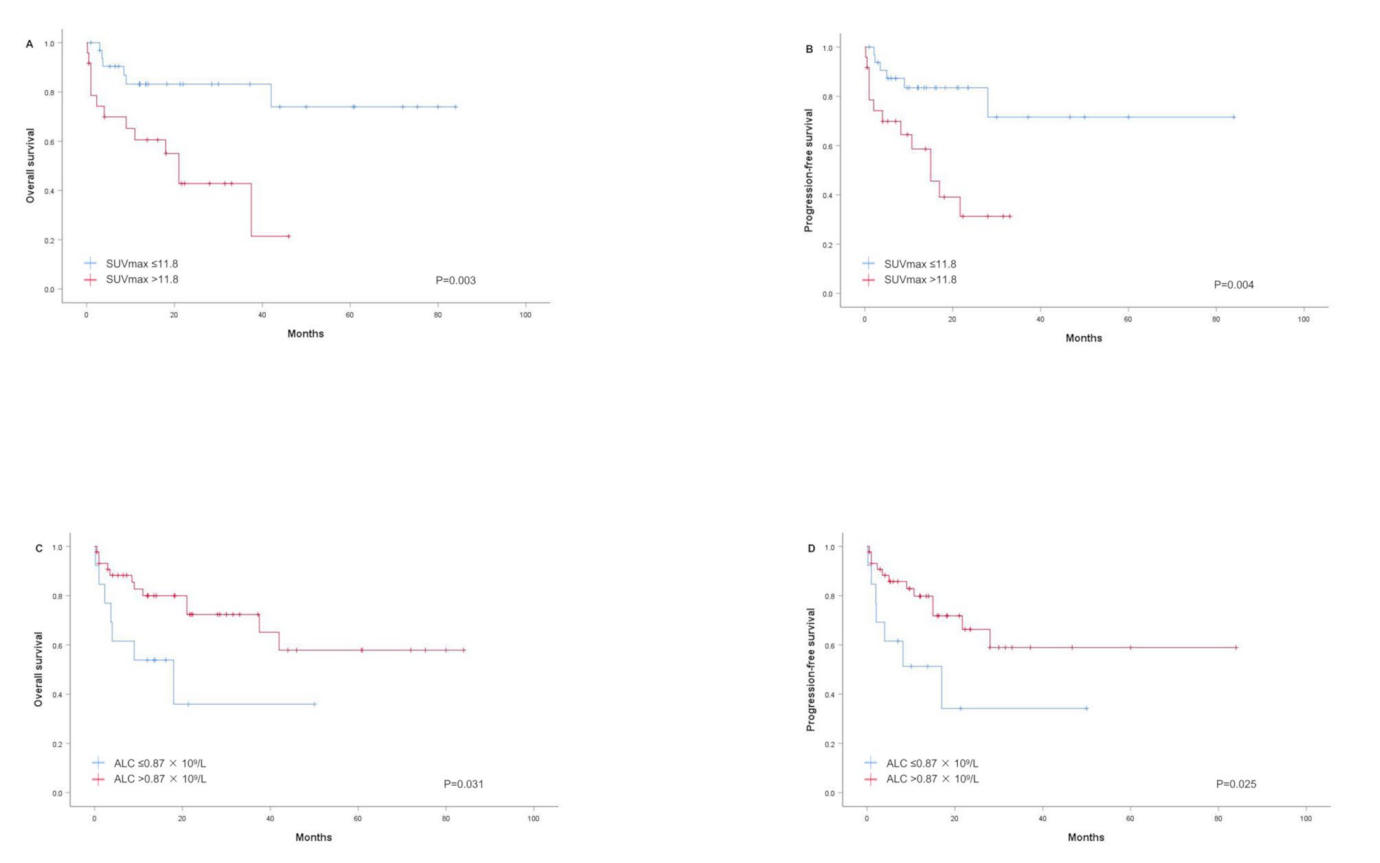
Kaplan-Meier curves for OS and PFS based on SUVmax and ALC in all patients. (**A**) OS in relation to SUVmax; (**B**) PFS in relation to SUVmax; (**C**) OS in relation to ALC; (**D**) PFS in relation to ALC. OS, overall survival; PFS, progression-free survival; SUVmax, maximum standardized uptake value; ALC, absolute lymphocyte count

The median follow-up duration was 13.8 months (range 0.2–84 months). Twenty-six (45.6%) patients had disease progression or relapse, and nineteen (33.3%) patients died. The 3-year OS and PFS rates for patients with high SUVmax were estimated to be 42.8% and 31.3%, respectively, compared to 83.2% and 71.6% for those with low SUVmax (*p* = 0.003 and 0.004, Fig. 2A and B). The 3-year OS and PFS rates for patients with high ALC were 72.3% and 58.9%, respectively, compared to 35.9% and 34.2% for those with low ALC (*p* = 0.031 and 0.025, Fig. 2C and D).

The results of the univariate analysis of clinical parameters are presented in Table 3. Adverse prognostic factors for both OS and PFS were included advanced Ann Arbor stage, IPI score > 2, PINK score ≥ 2, elevated LDH, distant lymph node involvement, pre-treatment SUVmax > 11.8, and pre-treatment ALC ≤ 0.87 × 10^9^/L. Additionally, NRI score and B symptoms were independent risk factors for PFS in ENKTCL patients.

**Table 3:** Univariate survival analyses of prognostic factors in ENKTCL

Characteristics	OS			PFS	
	HR (95% CI)	*P* value		HR (95% CI)	*P* value
Gender (male/female)	1.431(0.512–4.002)	0.494		1.213(0.436–3.374)	0.712
Age (> 60/≤60)	1.046(0.410–2.668)	0.925		1.076(0.423–2.736)	0.878
IPI score (≤ 2/>2)	4.162(1.650–10.500)	0.003		6.990(2.503–19.523)	0.000
NRI score (≤ 2/>2)	2.413(0.971–5.996)	0.058		2.581(1.033–6.453)	0.043
PINK score (< 2/≥2)	3.330 (1.310–8.463)	0.011		4.967 (1.758–14.037)	0.002
ECOG PS (< 2/≥2)	0.590 (0.078–4.442)	0.609		0.831 (0.107–6.427)	0.859
Ann Arbor stage (I-II/III-IV)	2.790(1.102–7.064)	0.030		3.363 (1.298–8.714)	0.013
B symptoms (yes/no)	2.411 (0.969–6.001)	0.059		2.688 (1.062−6.800)	0.037
Tumor location (UADT/EUADT)	1.182 (0.425–3.290)	0.749		1.341 (0.478–3.763)	0.577
Distant lymph node involvement (yes/no)	0.337 (0.133–0.850)	0.021		0.259 (0.097–0.691)	0.007
Albumin (< 35 g/L/≥35 g/L)	0.661 (0.266–1.642)	0.373		0.665 (0.2666–1.661)	0.382
EBV-DNA (positive/negative)	0.394 (0.112–1.383)	0.146		0.370 (0.105–1.304)	0.122
LDH (normal/elevated)	3.411 (1.222–9.516)	0.019		3.742 (1.334–10.498)	0.012
Ki−67 (< 70%/≥70%)	1.649 (0.618–4.401)	0.318		1.664 (0.623–4.439)	0.309
SUVmax (≤ 11.8/>11.8)	4.086 (1.514–11.025)	0.005		3.711 (1.409–9.778)	0.008
ALC (≤ 0.87 × 10^9^/L/>0.87 × 10^9^/L)	0.370 (0.143–0.954)	0.040		0.357 (0.139–0.918)	0.033
SUVmax-ALC	3.005 (1.558–5.798)	0.001		2.862 (1.514–5.410)	0.001

HR, hazard ratio; CI, confidence interval; OS, overall survival; PFS, progression-free survival; IPI, International Prognostic Index; NRI, nomogram-revised risk index; PINK, prognostic index of natural killer lymphoma; ECOG, Eastern Cooperative Oncology Group; PS, performance status; UADT, upper aerodigestive tract; EUADT, extra-upper aerodigestive tract; LDH, lactate dehydrogenase; EBV-DNA, Epstein-Barr virus DNA; SUVmax, maximum standardized uptake value; ALC, absolute lymphocyte count

As components of the PINK index are important and commonly used prognostic factors in ENKTCL, we included these components in the multivariate analysis along with pre-treatment SUVmax and ALC as dichotomized variables. The results indicated that pre-treatment SUVmax and ALC were independent predictors for OS (*p* = 0.027 and 0.043) and PFS (*p* = 0.039 and 0.049) (Table 4).

**Table 4:** Multivariate Cox proportional hazard regression analysis

Characteristics	OS			PFS	
	HR (95% CI)	*P* value		HR (95% CI)	*P* value
Model 1					
Age (> 60/≤60)	1.801(0.602–5.389)	0.293		1.909 (0.650–5.663)	0.238
Ann Arbor stage (I-II/III-IV)	1.414(0.429–4.662)	0.569		1.968 (0.629–6.156)	0.244
Tumor location (UADT/EUADT)	0.810 (0.234–2.807)	0.740		1.146 (0.341–3.849)	0.826
Distant lymph node involvement (yes/not)	0.516 (0.150–1.774)	0.294		0.417 (0.128–1.357)	0.146
SUVmax (≤ 11.8/>11.8)	3.553 (1.158–10.902)	0.027		3.035 (1.058–8.710)	0.039
Model 2					
Age (> 60/≤60)	1.468 (0.509–4.233)	0.477		1.669 (0.581–4.791)	0.341
Ann Arbor stage (I-II/III-IV)	1.979 (0.615–6.367)	0.252		2.654 (0.845–8.332)	0.094
Tumor location (UADT/EUADT)	0.744 (0.230–2.414)	0.623		1.009 (0.318–3.201)	0.988
Distant lymph node involvement (yes/not)	0.420 (0.1296–1.366)	0.149		0.393 (0.127–1.219)	0.106
ALC (≤ 0.87 × 10^9^/L/>0.87 × 10^9^/L)	0.365 (0.137–0.971)	0.043		0.378 (0.143–0.995)	0.049
Model 3					
Age (> 60/≤60)	1.708 (0.584–5.001)	0.329		1.931 (0.659–5.659)	0.230
Ann Arbor stage (I-II/III-IV)	1.501 (0.480–4.701)	0.485		2.094 (0.686–6.393)	0.194
Tumor location (UADT/EUADT)	0.840 (0.248–2.843)	0.779		1.233 (0.373–4.082)	0.731
Distant lymph node involvement (yes/not)	0.547 (0.166–1.803)	0.321		0.480 (0.151–1.528)	0.214
SUVmax-ALC	2.614 (1.303–5.246)	0.007		2.402 (1.224–4.715)	0.011

HR, hazard ratio; CI, confidence interval; OS, overall survival; PFS, progression-free survival; UADT, upper aerodigestive tract; EUADT, extra-upper aerodigestive tract; SUVmax, maximum standardized uptake value; ALC, absolute lymphocyte count

### The prognostic value of the SUVmax-ALC model

While pre-treatment SUVmax and ALC individually have prognostic significance, they have limited ability to identify patients in the high-risk category as single variables. Therefore, we developed a novel predictive model combining pre-treatment SUVmax and ALC as dichotomized variables to risk stratify ENKTCL patients. According to the prognostic model, patients were categorized into a low-risk group (SUVmax ≤ 11.8 and ALC > 0.87 × 10^9^/L), an intermediate-risk group (SUVmax ≤ 11.8 and ALC ≤ 0.87 × 10^9^/L, or SUVmax > 11.8 and ALC > 0.87 × 10^9^/L), and a high-risk group (SUVmax > 11.8 and ALC ≤ 0.87 × 10^9^/L). The ROC analysis of the SUVmax-ALC prognostic model for OS yielded an improved AUC of 0.714 ( 95% CI 0.567–0.861, *p* = 0.009) (Fig. 1C). The predictive performance of the combined model was superior to that of the single parameters (Fig. 1).

**Fig. 3:**
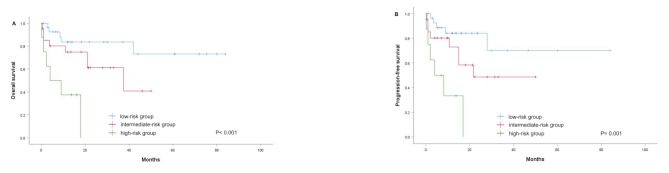
Kaplan-Meier methods were performed to estimate OS and PFS for the entire cohort of patients stratified by the SUVmax-ALC. OS, overall survival; PFS, progression-free survival; SUVmax, maximum standardized uptake value; ALC, absolute lymphocyte count

The associations between clinical features and SUVmax-ALC were evaluated. Patients with high-risk SUVmax-ALC were significantly associated with a high IPI score (*p* = 0.009) and tended to be related to poor ECOG PS (*p* = 0.062), advanced stage (*p* = 0.085), and B symptoms (*p* = 0.051) (Table 2). Comparison of survival curves showed a statistically significant difference in OS among the three groups (*p* < 0.001, Fig. 3A). The vast majority (23/28) of patients in the low-risk group survived, while three-quarters (6/8) of patients in the high-risk group died due to treatment failure. Thus, the overall survival of the high-risk group was significantly lower than that of the low-risk group. Consistent with their strong correlation with OS, these risk categories were also significantly associated with PFS (*p* = 0.001, Fig. 3B). Univariate analysis showed that the SUVmax-ALC prognostic model significantly impacted prognosis (OS: *p* = 0.001; PFS: *p* = 0.001) (Table 3). Multivariate analysis confirmed that SUVmax-ALC was an independent predictor for OS (*p* = 0.007) and PFS (*p* = 0.011) in patients with ENKTCL (Table 4).

### Comparison of the predictive accuracy for OS between the SUVmax-ALC and IPI, NRI, and PINK scores

**Fig. 4:**
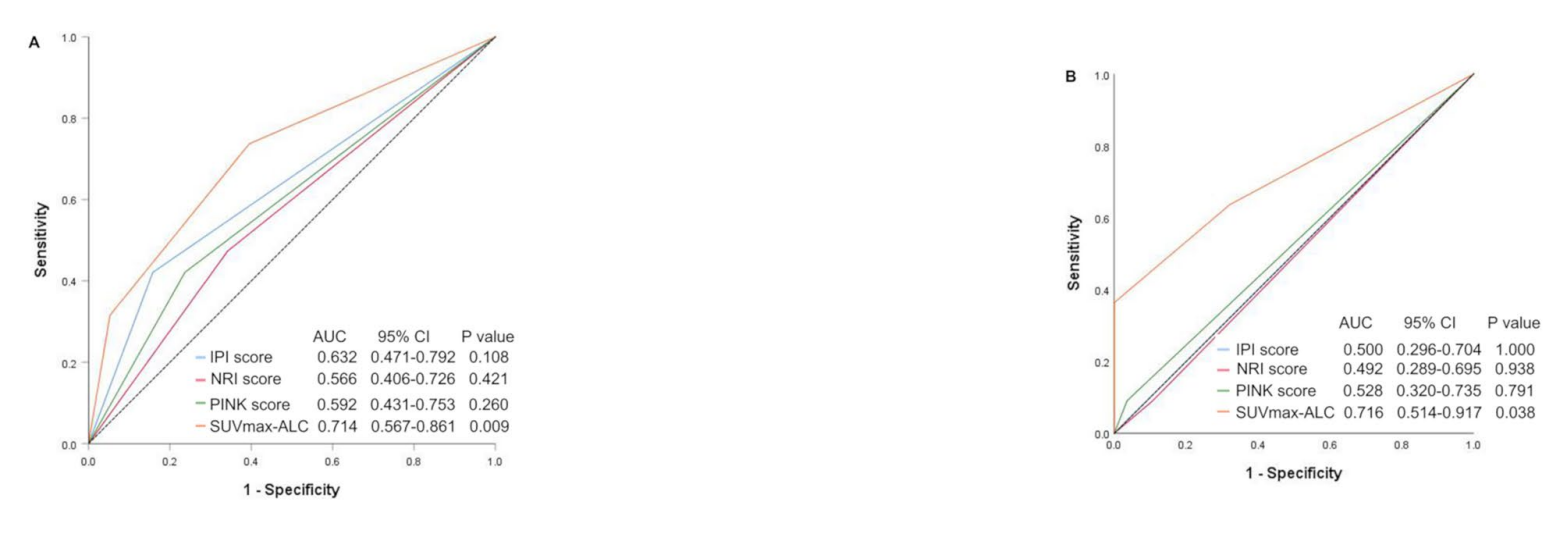
Comparison of the SUVmax-ALC. The SUVmax-ALC was compared with the IPI, NRI, and PINK by the AUC. The AUC for predicting OS for whole (**A**) and early-stage (**B**) patients. SUVmax, maximum standardized uptake value; ALC, absolute lymphocyte count; IPI, International Prognostic Index; NRI, nomogram-revised risk index; PINK, prognostic index of natural killer lymphoma; AUC, area under the curve; OS, overall survival

Kaplan-Meier analysis showed that the IPI, NRI, and PINK scores were good predictors of OS for the entire cohort (*p* = 0.001, 0.049, and 0.007, respectively), but could not distinguish between low-risk and high-risk groups in early-stage patients. We compared the OS prediction accuracy of the SUVmax-ALC model with the IPI, NRI, and PINK scores for the entire and early-stage cohorts. In the whole cohort, the SUVmax-ALC model demonstrated superior predictive accuracy for OS compared to the IPI, NRI, and PINK scores (Fig. 4A). Similarly, in the early-stage cohort, the SUVmax-ALC model outperformed the IPI, NRI, and PINK scores in predicting OS (Fig. 4B). Moreover, AUCs in the SUVmax-ALC model were consistently higher than others in the entire cohort and early-stage patients. According to the findings, SUVmax-ALC is a more precise and practical tool for classifying and separating OS in patients with all and early-stage ENKTCL.

### The SUVmax-ALC model identifies high-risk patients and provides additional prognostic information when superimposed on the IPI, NRI, and PINK scores

**Fig. 5:**
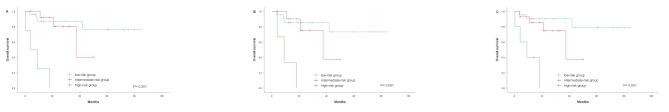
OS analysis. (**A**) OS based on risk stratification for patients in the IPI low-risk group by the SUVmax-ALC; (**B**) OS based on risk stratification for patients in the NRI low-risk group by the SUVmax-ALC; (**C**) OS based on risk stratification for patients in the PINK low-risk group by the SUVmax-ALC. OS, overall survival; SUVmax, maximum standardized uptake value; ALC, absolute lymphocyte count; IPI, International Prognostic Index; NRI, nomogram-revised risk index; PINK, prognostic index of natural killer lymphoma

Given that SUVmax-ALC is independent of the IPI, NRI, and PINK scores, we aimed to add the model into the IPI, NRI, and PINK scores to ascertain whether it may offer further predictive information when combined with these established scores. To evaluate this possibility, we stratified low-risk patients based on PINK score < 2, NRI score ≤ 2, and IPI score ≤ 2. Subgroup analyses demonstrated that SUVmax-ALC was associated with prognostic value in low-risk IPI groups. The 2-year OS in the low-, intermediate-, and high-risk groups based on SUVmax-ALC was 86.9%, 80.2%, and 0, respectively (*p* < 0.001) (Fig. 5A). In the IPI low-risk group, 4 patients (9.3%) were reclassified as high-risk according to the SUVmax-ALC model, and all died due to disease progression, with 3 patients surviving less than 1 year. Similarly, after adding SUVmax-ALC to the NRI low-risk group, the 2-year OS in the low-, intermediate-, and high-risk groups was 85.2%, 75.0%, and 0, respectively (*p* < 0.001) (Fig. 5B). Among these, 3 patients (8.6%) were reclassified as high-risk by the SUVmax-ALC model, all died due to disease progression, with 2 surviving less than 1 year. In addition, SUVmax-ALC showed a greater ability to distinguish differences in survival among patients in the low-risk PINK group. The 2-year OS in the low-, intermediate-, and high-risk groups was 90.2%, 74.5%, and 0, respectively (*p* < 0.001) (Fig. 5C). Of these patients, 5 patients (12.5%) were reclassified as high-risk by the SUVmax-ALC model, 4 died due to disease progression, with 3 surviving less than 1 year.

### Prognostic performance of SUVmax-ALC in patients received non-anthracycline-based regimens

**Fig. 6:**
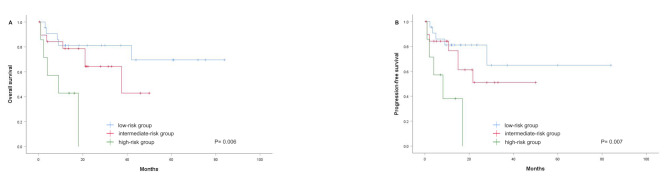
Kaplan-Meier methods were performed to estimate OS and PFS for patients treated with non-anthracycline chemotherapy regimens stratified by the SUVmax-ALC. OS, overall survival; PFS, progression-free survival; SUVmax, maximum standardized uptake value; ALC, absolute lymphocyte count

We further evaluated the performance of the SUVmax-ALC model in a subset of patients who received non-anthracycline-based regimens (*n* = 49). As shown in Fig. 6, the SUVmax-ALC model demonstrated significant prognostic value in this cohort, with a 2-year OS of 81.1%, 64.3%, and 0 (*p* = 0.006) (Fig. 6A) and a 2-year PFS of 81.1%, 51.0%, and 0 (*p* = 0.007) (Fig. 6B) in the low-, intermediate-, and high-risk groups, respectively.

In this study, interim PET/CT scans were conducted in 14 of the 49 patients following 3 to 6 cycles of chemotherapy. Specifically, 3 patients underwent interim PET/CT after 3 cycles, 7 patients after 4 cycles, 2 patients after 5 cycles, and 2 patients after 6 cycles. The risk stratification method based on pre-treatment SUVmax-ALC (pre-SUVmax-ALC) was utilized for interim SUVmax-ALC assessment. Comparing the interim SUVmax-ALC model with the pre-SUVmax-ALC model revealed that 2 patients initially classified as high-risk by pre-SUVmax-ALC were reclassified as intermediate-risk by interim SUVmax-ALC, while 2 patients initially classified as low-risk by pre-SUVmax-ALC were also reclassified as intermediate-risk. Additionally, 2 patients initially classified as intermediate-risk by pre-SUVmax-ALC were reclassified as low-risk based on interim SUVmax-ALC results. Notably, 8 of the 14 patients (57.1%) demonstrated consistent risk stratification between the pre-SUVmax-ALC and interim SUVmax-ALC models.

End-of-treatment PET/CT scans were performed in 7 patients after completing their first-line therapy. There were 4 patients underwent these scans after 4 cycles of chemotherapy, 1 patient after 5 cycles, and 2 patients after 6 cycles. The pre-SUVmax-ALC risk stratification method was also applied to assess end-of-treatment SUVmax-ALC, 1 patient initially classified as high-risk by pre-SUVmax-ALC was reclassified as intermediate-risk by end-of-treatment SUVmax-ALC. In 6 of the 7 patients (85.7%), the pre-SUVmax-ALC model was consistent with end-of-treatment SUVmax-ALC risk stratification.

## Discussion

ENKTCL is an extremely aggressive subtype of non-Hodgkin lymphoma. Although most patients are diagnosed early in the disease, the reported 5-year OS rate is less than 60% [10, 28–31]. Therefore, it is very important to evaluate and predict the prognosis of patients accurately.

The parameters derived from ^18^F-FDG PET/CT serve as valuable diagnostic and staging tools for patients with ENKTCL [32]. The SUVmax is a commonly utilized metabolic indicator reflecting tumor aggressiveness. Several studies have demonstrated that the pre-treatment SUVmax can predict patient prognosis [17. 18, 20–22]. However, the optimal threshold for SUVmax remains undetermined, with varying data reported in the literature. For instance, Chang et al. [18]. analyzed 52 ENKTCL cases and concluded that a pre-treatment SUVmax > 15.1 indicated a poor OS independent of other clinical characteristics, though it did not correlate with PFS. In contrast, Kim et al. [22] retrospectively examined 20 ENKTCL patients and observed that a high SUVmax (> 8.1) was significantly linked to poor PFS but not OS. Similarly, Bai et al. [21] conducted a retrospective analysis on 81 ENKTCL patients, finding that a pre-treatment SUVmax > 15 was indicative of a poor prognosis in newly diagnosed cases. We thought the difference in the SUVmax cut-off value may be multifactorial. The discrepancies in the SUVmax cut-off values across these studies suggest a multifactorial origin, necessitating local validation of the cut-off value. Nonetheless, a higher tumor SUVmax generally correlates with more aggressive disease and poorer prognosis. Our finding revealed that a pre-treatment SUVmax > 11.8 was a poor predictor for both OS and PFS. Additionally, elevated SUVmax was significantly associated with adverse tumor characteristics, including high IPI score, advanced stage, and elevated LDH levels, which is consistent with previous research [20, 21]. Ki-67, a well-established marker of tumor proliferation [33], was found by Liu et al. [34]. to be an unfavorable prognostic factor for OS in advanced ENKTCL when its expression exceeded 70%. In our study, we identified a significant correlation between SUVmax and Ki-67, suggesting that SUVmax could potentially assess tumor cell proliferation, with higher values indicating more aggressive biological behavior and rapid tumor growth [21].

To more accurately estimate OS and PFS and to optimize individualized monitoring strategies for patients with ENKTCL, we propose that integrating the SUVmax with conventional clinical parameters can enhance prognostic stratification for ENKTCL patients. ALC serves as a surrogate marker of immune homeostasis, and lymphopenia is recognized as an indicator of immune insufficiency. Huang et al. [26]. reported that patients with low ALC exhibited significantly worse PFS and OS. Our univariate and multivariate analyses also confirmed that ALC is an independent prognostic factor for ENKTCL patients. In the present study, we developed a novel prognostic model, the SUVmax-ALC index, which combines oncological and immunological aspects to predict PFS and OS in newly diagnosed ENKTCL patients. According to this model, ENKTCL patients were categorized into three distinct risk groups, each with significant differences in survival outcomes. The high-risk group was associated with poorer OS and PFS and exhibited adverse characteristics such as high IPI scores and B symptoms.

ENKTCL is characterized by aggressive behavior and poor prognosis. Consequently, several prognostic models, including the IPI, NRI, and PINK scores, have been proposed for risk assessment and treatment guidance. However, due to the uneven distribution of patients in different risk categories, a significant proportion of the population continues to exhibit poor prognosis despite appropriate treatment [10]. Therefore, there is a critical need to develop new prognostic factors for ENKTCL to accurately stratify patients for optimal personalized management. The SUVmax-ALC model, an independent prognostic factor from these established models, has demonstrated superior prediction results and provided additional prognostic information when integrated with IPI, NRI, and PINK scores. Specifically, the SUVmax-ALC model identified approximately 10% of patients as high-risk, particularly those with low-risk IPI, NRI, and PINK scores. This indicates that the SUVmax-ALC model can identify high-risk populations that the IPI, NRI, and PINK scores fail to detect, thereby enhancing clinical management.

The treatment of ENKTCL varies based on the patient’s condition, and currently, non-anthracycline-based chemotherapy is the mainstay of clinical practice. However, due to the heterogeneity of the disease, some newly diagnosed patients experience disease progression, and the survival outcomes for patients with relapsed or refractory ENKTCL remain unsatisfactory [10, 29, 30]. In this study, the majority of ENKTCL patients received non-anthracycline chemotherapy regimens, with 76.5% undergoing pegaspargase/l-asparaginase-based therapy. Our findings suggest that the SUVmax-ALC prognostic model remains predictive in patients receiving non-anthracycline-based chemotherapy regimens and can help identify patients with poor survival outcomes.

PET/CT is widely used in clinical practice and trials, as recommended by international consensus guidelines [35], to assess the effectiveness of first-line treatments and detect potential disease progression. In our study, only a limited number of patients underwent PET/CT examinations during the interim and at end of treatment. Nevertheless, the interim SUVmax-ALC and end-of-treatment SUVmax-ALC models demonstrated strong consistency with the pre-SUVmax-ALC model in terms of risk stratification, particularly the end-of-treatment SUVmax-ALC model. Due to the restricted sample size, further statistical analyses were not practicable. Future investigations with larger cohorts are necessary to validate these findings. Nonetheless, our pre-treatment prediction model appears to be suitable for integration into current clinical practice.

Despite the significant association between the SUVmax-ALC prognostic model and prognosis, this study has some limitations. First, the number of patients in this study was comparatively small because the incidence of ENKTCL is much lower than other non-Hodgkin lymphoma subtypes. Second, our study was a retrospective study conducted at a single center, so there may be potential sources of selection bias. Therefore, it is necessary to validate our findings in future large-scale prospective studies.

## Conclusion

In conclusion, the pre-treatment SUVmax and ALC are helpful in predicting the prognosis of patients with ENKTCL. In this study, we established the first model to predict prognosis of PFS and OS in ENKTCL patients using tumor metabolism and individual immune status. The pre-treatment SUVmax-ALC model showed a good predictive ability for risk stratification compared to IPI, NRI, and PINK scores, indicating that the pre-treatment SUVmax-ALC had considerable potential as an effective personalized prognostic tool for patients with ENKTCL.

## Data Availability

No datasets were generated or analysed during the current study.

## Abbreviations

ALC: Absolute lymphocyte count
AUC: Area under the curve
CI: Confidence interval
DLBCL: Diffuse large B cell lymphoma
EBV-DNA: Epstein-Barr virus DNA
ECCR: Ethics committee in clinical research
ECOG: Eastern Cooperative Oncology Group
ENKTCL: Extranodal natural killer/T-cell lymphoma
EUADT: Extra-upper aerodigestive tract
^18^F-FDG PET/CT: ^18^F-fluorodeoxyglucose positron emission tomography/computed tomography
HR: Hazard ratio
IPI: International Prognostic Index
LDH: Lactate dehydrogenase
MTV: Metabolic tumor volume
NHL: Non-Hodgkin lymphoma
NRI: Nomogram-revised risk index
OS: Overall survival
PFS: Progression-free survival
PINK: Prognostic index of natural killer T-cell lymphoma
ROC: Receiver operating characteristic
SUVmax: Maximum standardized uptake value
TLG: Total lesion glycolysis
UADT: Upper aerodigestive tract
VOI: Volume of interest
WHO: World Health Organization
